# Phenolic Profile and Antioxidant Capacity of Invasive *Solidago canadensis* L.: Potential Applications in Phytopharmacy

**DOI:** 10.3390/plants14010044

**Published:** 2024-12-26

**Authors:** Mirela Uzelac Božac, Danijela Poljuha, Slavica Dudaš, Josipa Bilić, Ivana Šola, Maja Mikulič-Petkovšek, Barbara Sladonja

**Affiliations:** 1 Department of Agriculture and Nutrition, Institute of Agriculture and Tourism, Karla Huguesa 8, 52440 Poreč, Croatia; mirela@iptpo.hr (M.U.B.); barbara@iptpo.hr (B.S.); 2 Agricultural Department, Polytechnic of Rijeka, Karla Huguesa 6, 52440 Poreč, Croatia; sdudas@veleri.hr; 3 METRIS Research Centre, Istrian University of Applied Sciences, Zagrebačka 30, 52100 Pula, Croatia; jbilic@iv.hr; 4 Department of Biology, Faculty of Science, University of Zagreb, 10000 Zagreb, Croatia; ivana.sola@biol.pmf.unizg.hr; 5 Department of Agronomy, Biotechnical Faculty, University of Ljubljana, Jamnikarjeva 101, 1000 Ljubljana, Slovenia; maja.mikulic-petkovsek@bf.uni-lj.si

**Keywords:** Canadian goldenrod, flower, invasive plants, leaf, plant extracts

## Abstract

Canadian goldenrod *(Solidago canadensis* L.), an invasive plant in Europe, is known for its allelopathic activity and is rich in bioactive compounds like flavonoids and phenolic acids, with significant pharmacological potential. This study presents the LC-MS phenolic profiles of leaf and flower extracts from *S. canadensis*, an invasive alien plant in the Istria region (Croatia). Total phenolics (TP) (45.78–110.68 mg GAE/g DW) and non-flavonoids (TNF) (28.38–72.20 mg GAE/g DW) were found to be more abundant in ethanolic than in methanolic extracts. The antioxidant capacity (AC), as measured by ABTS, DPPH, and FRAP assays, was higher in flower extracts compared to leaf extracts. A non-targeted metabolomics approach was used, and 41 phenolic compounds in leaves and 36 in flowers were identified, with hydroxycinnamic acids and flavonols being the most abundant. 5-caffeoylquinic acid was quantitatively predominant in the leaf extracts, while quercetin-3-rutinoside dominated the flower extracts. Five leaf-specific compounds were identified (dicaffeoylquinic acid 2, 4-*p*-coumaroylquinic acid 1, *p*-hydroxybenzoic acid, quercetin-3-rhamnoside, and quercetin acetylhexoside 1), suggesting targeted extraction for different pharmacological applications. This study highlights the therapeutic potential of *S. canadensis* and underscores the need for further research on the bioavailability, efficacy, and safety of its compounds, potentially transforming this ecological threat into a valuable resource for drug development.

## 1. Introduction

Plants have a long history of use in folk herbal medicine as food sources and remedies for treating various diseases. Their bioactive compounds, such as alkaloids, terpenoids, coumarins, nitrogen-containing and organosulfur compounds, and phenolics, are currently being researched to discover and develop new pharmaceuticals [1]. Phenolics, in particular, are significant due to their role as effective antioxidants and antibacterials [2]. They are synthesized through the shikimate and phenylpropanoid pathways, which convert primary metabolites like phenylalanine into biologically active phenolics such as quercetin, rutin, and chlorogenic acid. The shikimate pathway transforms simple carbohydrates into aromatic amino acids like phenylalanine and produces precursors like chorismate, which leads to phenolic acids such as caffeic acid, chlorogenic acid, and ferulic acid. The phenylpropanoid pathway converts phenylalanine into *p*-coumaric acid, a key intermediate for synthesizing flavonoids and polyphenols like quercetin, rutin, and epigallocatechin [3]. These compounds are known for their strong antioxidant, anti-inflammatory and antimicrobial activities [2]. Additionally, plant phenolics exhibit significant antitumor activity, inhibiting cancer initiation, progression, and metastasis in both in vitro and in vivo studies. Phenolics exert their effects by modulating cellular pathways such as growth factor-receptor interactions and signaling cascades (e.g., kinases and transcription factors), leading to cell cycle arrest, apoptosis, and reduced cell survival [4].

Phenolics in invasive alien plants are especially intriguing because some studies suggest they produce higher quantities of these compounds than native species [5] or the same species in their native ranges [6]. One theory is that the novel biochemistry of invasive plants in new habitats represents their response to new environments and climate conditions [7,8].

Canadian goldenrod, *Solidago canadensis* L., a perennial herb native to North America, was introduced in Europe in the early 17th century as an ornamental plant [9]. The species is a member of the *Solidago* genus (*Compositae*, *Asterales*), commonly referred to as “goldenrods” [10]. It quickly naturalized, spreading along abandoned fields and riverbanks. Nowadays, *S. canadensis* is widespread across Europe and is listed on the EPPO list of invasive alien plants [11]. Its invasive nature is enhanced by the fast vegetative propagation through long rhizomes, high growth rate, and allelopathy, which leads to the transformation of soil properties and plant communities [7,12,13]. *S. canadensis* exhibits strong allelopathic effects by releasing allelochemicals that inhibit the growth, germination, and development of neighboring plants, thereby reducing biodiversity and outcompeting native species. These allelochemicals are released through root exudates, plant residue decomposition, and leachates, which alter soil microbial communities and can degrade arbuscular mycorrhizal fungi, vital for nutrient and water uptake in native plants [6]. The phytotoxicity of *S. canadensis* is linked to oxidative stress and damage to cell membranes, leading to electrolyte leakage, reduced chlorophyll content, and impaired photosynthesis. Some of the key phenolic allelochemicals in this species that contribute to its allelopathic impact are chlorogenic acid, rutin (quercetin-3-O-rutinoside), kaempferol-3-O-D-glucoside, and quercitrine [6]. Phenolic allelochemicals interfere with several enzymes and the major physiological processes, such as phytohormone activity, mineral uptake, plant water balance and stomatal function, photosynthesis, respiration, organic synthesis of certain compounds, and flow of carbon, contributing to the phytotoxicity [14,15]. These phenolic compounds enhance species invasiveness but may offer potential as natural herbicides. The beneficial aspects of *S. canadensis* have been recognized for centuries. The herbal material of this invasive species has been used in European phytotherapy for a long time to treat urinary and genital diseases [16]. This species is well-known in traditional medicine due to its complex composition of specialized metabolites, such as polyphenolics, which contribute to its antioxidant, antimicrobial, and anti-inflammatory activities [16,17,18,19,20].

In our recent comprehensive review of *S. canadensis* phytochemicals [21], we highlighted the existing knowledge gaps, and the present study builds on this by providing the site-specific comparison of phenolic profiles between leaf and flower extracts of this species, further advancing our understanding of its potential for phytopharmaceutical applications. In this paper, we selected *S. canadensis*, commonly found in anthropogenically disturbed areas and along abandoned riverbanks in Istria, Croatia, for the first site-specific phytochemical screening to explore its potential for phytopharmaceutical applications. This choice was based on the understanding that phytochemical profiles are influenced by ecological and climatic conditions [22]. This study aimed to identify the main phenolic compounds and the antioxidant capacity of leaf and flower extracts of *S. canadensis* from Istria. For the first time, we provide a novel comparison of the phytochemical profile and antioxidant activity of individual parts of the plant—leaf and flower—offering a detailed insight into the significant differences between them. This analysis not only establishes a foundation for understanding the local biological activity of *S. canadensis* but also sets the stage for proposing a site-specific model of its exploitation as a provider of new ecosystem services. Alongside future ecological and biological analyses, we expect such a model to trigger similar approaches in broader geographical contexts.

For this purpose, we (1) spectrophotometrically measured the content of different groups of bioactive compounds (total phenolics, flavonoids, and non-flavonoids) and their antioxidant capacity using three standard assays (DPPH, ABTS, and FRAP); (2) identified and quantified the main phenolic compounds using the LC-DAD-MS method; and (3) statistically determined the influence of different factors, such as plant part (leaf and flower) and solvent (70% ethanol and 80% methanol), on measured variables using two-way ANOVA and Tukey’s test (*p* ≤ 0.05 and 0.01).

## 2. Results and Discussion

### 2.1. Phenolic Content and Antioxidant Capacity

**Figure 1:**
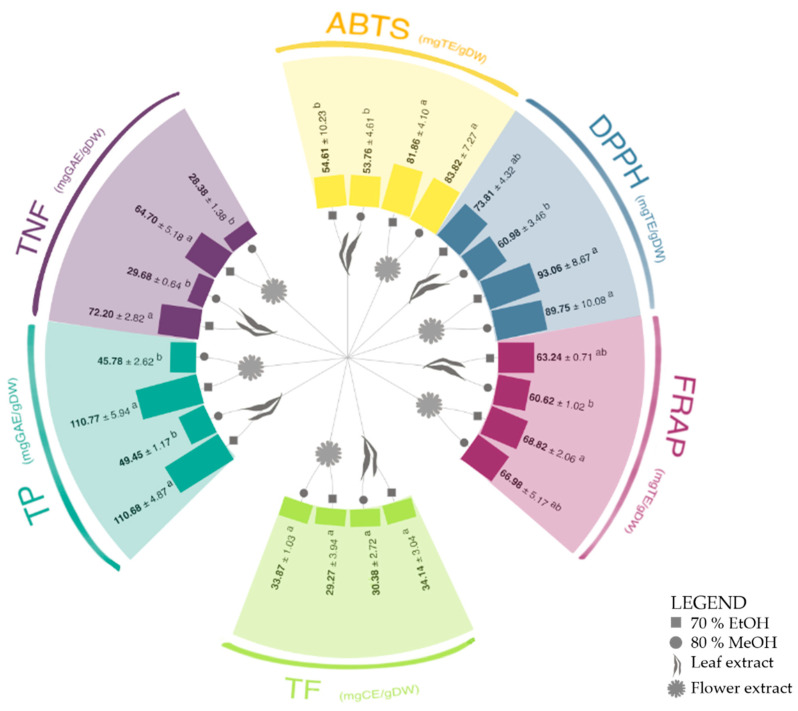
The content of total phenolics (TP), total non-flavonoids (TNF), and total flavonoids (TF), and antioxidant capacity measured by ABTS, DPPH, and FRAP assays in *S. canadensis* leaf and flower extracts in 70% ethanol and 80% methanol. Different letters (a–b) in the same section indicate significant differences between the measured values (two-way ANOVA, Tukey’s test, *p* ≤ 0.01). All values are also shown in Table S1.

The type of solvent had a significant effect on total phenolic (TP) and total non-flavonoids (TNF) content in tested extracts, with values being higher for ethanol (EtOH) than for methanol (MeOH) (Figure 1, Table S1). The plant part significantly affected TNF content, which was higher in leaf than in flower extracts, but only when the EtOH was used as a solvent. Neither the plant part nor the solvent significantly influenced the total phenolic (TP), total flavonoid (TF), or total non-flavonoid (TNF) content. The highest TP values were found in the ethanolic extracts of leaves and flowers, measuring 110.68 and 110.77 mg of gallic acid equivalent (GAE)/g of dry weight (DW), respectively. The TNF values were higher in the ethanolic extract for both leaves and flowers, with 72.20 mg of GAE/g DW (in leaves) and 64.70 mg of GAE/g DW (in flowers), compared to the methanolic extracts, which contained 29.68 and 28.38 mg of GAE/g DW in leaves and flowers extracts, respectively. This could be attributed to the solvent’s efficiency in solubilizing specific non-flavonoid compounds. Previous studies reported that the choice of solvent depends on the extraction method and the type of phenolic compounds desired, with methanol being optimal for maceration and microwave extraction of hydrolyzable tannins and polyphenols, while ethanol is best for infusion and the extraction of condensed tannins across most methods [23].

The TF values were similar across solvents and plant parts, ranging from 29.27 to 34.14 mg of catechin equivalent (CE)/g DW.

The TP and TF contents of *S. canadensis* leaf extract obtained in our study were higher than in the findings of Du et al. [24], who analyzed samples collected in China, where this species is also invasive. Furthermore, flower extracts exhibited significantly higher TP and TF content compared to the values reported by Shelepova et al. [25]. Their study determined TP and TF contents of 105.36 ± 1.45 mg GAE/100 g and 58.23 ± 0.17 mg quercetin equivalent (QE)/100 g, respectively, in 96% EtOH and 80% MeOH extracts. The higher levels of detected flavonoids can be attributed to variations in climatic conditions and vegetative phase [26,27]. In clay soils, plant fixation of NH_4_^+^ increases with increasing pH [28]. In soils with a high pH, the roots of *S. canadensis* secrete more compounds from the flavonoid group compared to other phenolic groups. This aligns with the fact that our samples were collected along riverbanks in Istria with alkaline swamp-clay soil on a sunny day in August.

The antioxidant capacity (AC) of *S. canadensis* flower extracts, measured across all three assays, was higher than that of the leaf extracts, indicating that the plant part had a more significant effect on the AC than the solvent used. AC of *S. canadensis* was also tested by other authors, who proved the antioxidant capacity of aerial parts [17,19,20,29,30,31] and root [20,32] extracts of this species. However, comparing the obtained values is challenging due to the differences in the extraction methods, solvents used, and the tests themselves. Deng et al. [19], for example, showed that the AC and contents of TP, TF, and total tannins depended on the ripeness stage, tissue type, and extraction method. Furthermore, a comprehensive evaluation of antioxidant potential requires multiple in vitro assays due to the diverse mechanisms by which antioxidants operate. Direct comparisons between methods are thus challenging [33].

**Figure 2:**
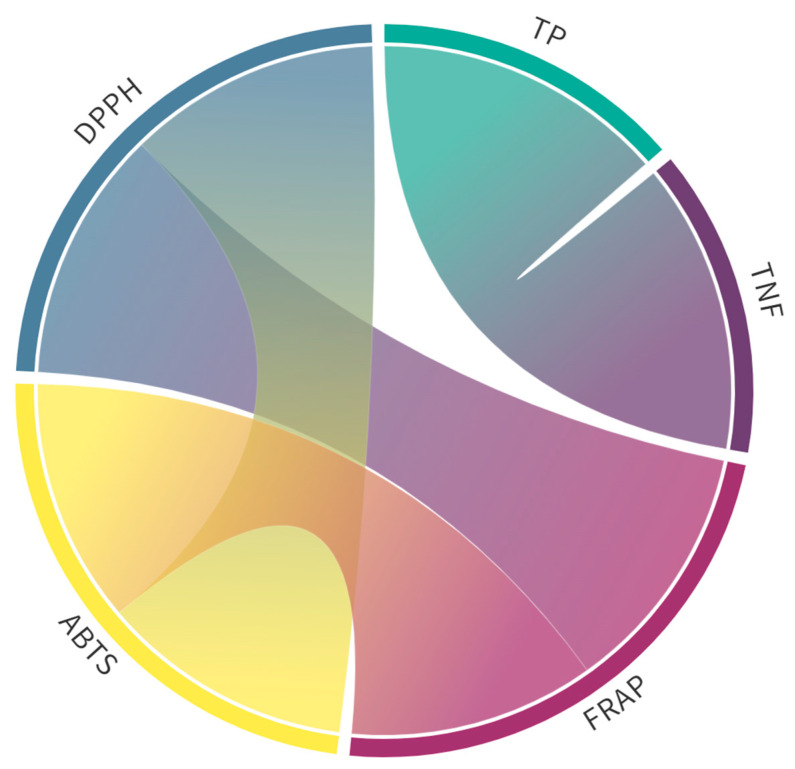
Pearson’s correlation coefficients of total phenolics (TP) and non-flavonoids (TNF) contents and antioxidant capacities in extracts in both solvents, determined by ABTS, DPPH, and FRAP assays. Only significant correlations (*p* ≤ 0.01; Table S2) are shown. The outer ring represents the variables (TP, TNF, DPPH, ABTS, and FRAP), while the connecting bands show the strength of correlations between the variables: thicker bands represent stronger correlations.

In all the extracts, highly significant (*p* ≤ 0.01) total (0.9 < r < 1.0) and very strong positive (0.7 < r < 0.9) correlations, according to the Roemer-Orphal scale, were observed between AC values determined by ABTS, DPPH, and FRAP assays, as well as between TP and TNF values (Figure 2). There were no other significant correlations (Table S2).

### 2.2. Identification and Quantification of Phenolic Compounds

**Figure 3:**
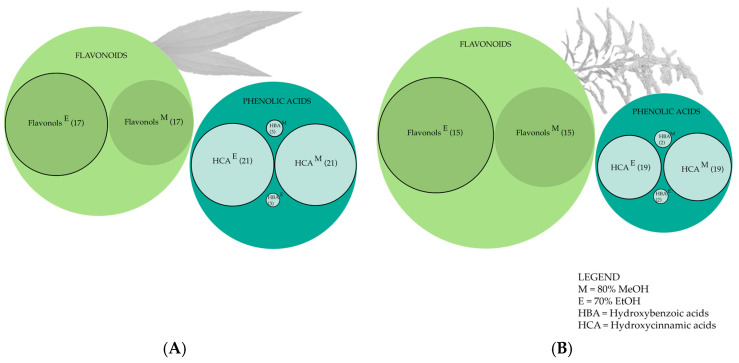
The main phenolic groups obtained by the LC-DAD-MS method in the leaf (**A**) and flower (**B**) extracts. The ratio of the circles’ sizes corresponds to the total concentration of individual phenolic groups (mg/g of dry weight (DW)), and the numbers in parentheses indicate the numbers of identified individual compounds within each phenolic group. The circle border indicates statistically significant differences between concentrations of individual phenolic groups in the plant part (two-way ANOVA, Tukey’s test, *p* ≤ 0.01).

We identified 41 phenolic compounds in leaf and 36 in flower extracts, belonging to the groups of phenolic acids (hydroxycinnamic and hydroxybenzoic acid derivatives) and flavonoids (flavonols) (Table 1, Figure 3). In leaf extracts, flavonols dominated, accounting for 55% of the total detected phenolics in ethanolic and 60% in methanolic extracts (Figure 3A). Similarly, the flower extracts had the highest share of flavonols (76% in EtOH and 67% in MeOH) (Figure 3B).

Leaf extracts exhibited the highest compound diversity within the hydroxycinnamic acids (HCA) group (21 compounds), followed by flavonols (17 compounds) (Table 1, Figure 3A). The flower extracts showed a similar distribution of phenolic groups; HCA was represented by 19 compounds, followed by flavonols, represented by 15 compounds (Table 1, Figure 3B).

These findings are consistent with the results reported in our recent study on *S. canadensis* phytochemicals, which provides a more detailed comparison of the phytochemical composition of this species and highlights hydroxycinnamic and hydroxybenzoic acids, as well as flavonoids, as the primary polyphenolic constituents [21]. As noted in several studies, hydroxycinnamic acids, particularly chlorogenic acid, are the most prevalent phenolic acids, with flavonols, including quercetin and kaempferol, being the dominant flavonoids found in various plant parts [6,31]. These compounds were also noted in the flowers and leaves of *S. canadensis*, with significant differences in the glycoside composition—rutin being the most abundant in flowers and hyperoside in leaves [34]. Our study similarly identifies a rich diversity of hydroxycinnamic acids in both leaves and flowers, supporting the conclusions of Woźniak et al. [31], who reported a high concentration of chlorogenic acid derivatives in aerial parts. Notably, we also observe that *S. canadensis* contains a broad spectrum of flavonoids, with flavonols being the predominant group, consistent with previous reports [6,17]. Additionally, our data on the comparison of different plant parts complements the findings by Woźniak et al. [31], where the underground parts were enriched with hydroxycinnamic acid conjugates, highlighting distinct biochemical profiles between the aerial and underground components of *S. canadensis*. These observations further underline the potential for *S. canadensis* to be explored for its diverse bioactive compounds in both ecological and pharmacological contexts. Five compounds were leaf-specific (dicaffeoylquinic acid 2, 4-*p*-coumaroylquinic acid 1, *p*-hydroxybenzoic acid, quercetin-3-rhamnoside, and quercetin acetylhexoside 1), while we found no flower-specific compounds. Our results differ from the results of Zekič et al. [35], who detected dicaffeoylquinic acid in the *S. canadensis* inflorescence from Slovenia.

Various factors significantly affected the content of different phenolic groups (*p* ≤ 0.01), whereas for total phenolics, the significance was observed at the *p* ≤ 0.05 level. The plant part had a significant impact on the HCA content, while both the solvent and plant part affected the total hydroxybenzoic acids (HBA) content. HCAs might be more concentrated in certain plant parts due to the specific biosynthetic pathways or storage mechanisms in those parts. The higher concentration of HCAs in leaves compared to flowers can be explained by several factors; leaves are often more exposed to herbivores, pathogens, and environmental stress (like UV radiation) than flowers. HCAs, which have antioxidant, antimicrobial, and UV-absorbing properties, play a crucial role in protecting leaves from these threats. Plants may, therefore, synthesize and accumulate more HCAs in leaves as a protective measure [36]. Leaves are the primary site of photosynthesis, a process that generates reactive oxygen species (ROS) as byproducts. With their antioxidant properties, HCAs help neutralize ROS, protecting the leaf tissues from oxidative damage. The high metabolic activity in leaves may necessitate higher levels of HCAs [37]. The biosynthesis of HCAs occurs through the phenylpropanoid pathway, which is active in many plant parts but is particularly prominent in leaves due to their role in photosynthesis and defense. This pathway is closely linked to the production of lignin, flavonoids, and other phenolics, which are often more concentrated in leaves [38,39]. Both the plant part and the solvent significantly influenced the flavonol content (two-way ANOVA and Tukey’s test at *p*-value ≤ 0.01). One of the reasons is assumed to be their structure. Namely, flavonols (a type of flavonoid) are more structurally complex than HCAs and HBAs. They contain multiple hydroxyl groups attached to a polycyclic ring system, making them more reactive to changes in extraction conditions, such as the solvent used and the plant part. They often bind to sugars, proteins, or other cell wall components, forming complex molecules such as glycosides. The ability of a solvent to break these bonds can vary significantly, affecting how much flavonol is extracted from different plant parts [36,40]. In our case, the influence of the solvent on the flavonoid content was significant only in the flower extracts, with deviations shown for individual compounds. HCAs and HBAs tend to be less bound to complex matrices and are more likely to exist in free forms or as esters that are easier to extract. As a result, solvent choice may not have as significant an impact on their extraction [41]. Flavonols are often unevenly distributed among different plant parts. For instance, leaves tend to have higher concentrations due to their role in protecting the plant from UV radiation and oxidative stress. This variation in distribution among plant parts means the part chosen for extraction can significantly impact the flavonol content [42,43]. HCAs and HBAs are more evenly distributed across various plant tissues, being involved in general plant metabolism and cell wall structure. Therefore, the specific plant part used for extraction might not result in such pronounced differences in HCA or HBA content compared to flavonols [39]. Flavonols are part of the flavonoid biosynthesis pathway, which can be more sensitive to environmental factors such as light exposure, stress, and plant part-specific metabolic activity. This means that the location within the plant and the extraction conditions can have a more significant impact on flavonol levels [44,45]. HCAs and HBAs, on the other hand, are produced through the shikimate pathway, which tends to be more stable across different plant parts and less influenced by environmental conditions, leading to relatively consistent levels across plant parts and solvents [39,46].

**Figure 4:**
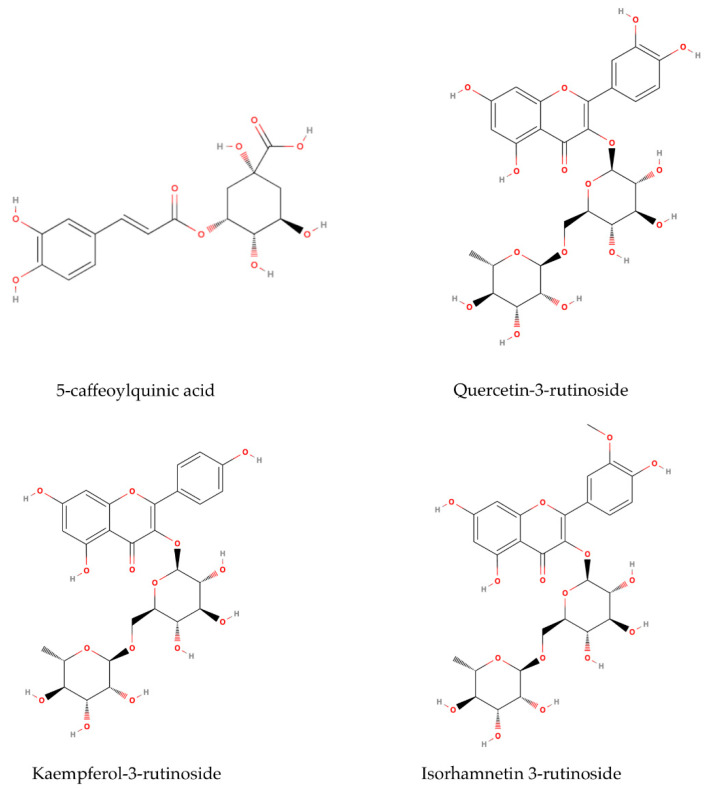
The structural molecules of four main compounds found in leaf and flower extracts in *S. canadensis* (MolView. Available online: https://molview.org/, accessed on 11 August 2024).

In the leaf extracts, 5-caffeoylquinic acid 1 was the major compound in both ethanolic and methanolic extracts (17.75 mg/g DW and 20.42 mg/g DW, respectively) (Table 1). The 5-*O*-caffeoylquinic acid (5-CQA) (Figure 4), also known as chlorogenic acid, is one of the major chlorogenic acids present in many fruits, vegetables, and herbs [47]. This compound exhibits antioxidant activity against oxidative stress-mediated liver injury, as well as anti-inflammatory and antimicrobial capacity [47,48]. These properties make 5-CQA potentially useful as a preservative in the food, pharmaceutical, and cosmetic industries. Marksa et al. [18] discovered that chlorogenic acid was the critical component responsible for the antioxidant properties in the leaves and inflorescences of the sibling species *S. gigantea*. In contrast, in *S. canadensis*, the primary antioxidant component was 3,5-dicaffeoylquinic acid. Their study revealed that di-caffeoylquinic acids have a stronger radical scavenging effect than mono-caffeoylquinic acids. Similar major compounds, including chlorogenic acid, quercitrin, and rutin, were identified in 70% ethanolic extracts of the leaves and inflorescences of *S. canadensis* [49]. Chlorogenic acid, quercetin, and kaempferol rutinosides were identified as the main compounds in both aerial and underground parts in 70% methanol *S. canadensis* and *S. gigantea* extracts [31]. Furthermore, Shelepova et al. [25] identified phenolic acids such as chlorogenic, caffeic, and ferulic acids as the main components in the *S. canadensis* flower extracts.

Quercetin-3-rutinoside, also known as rutin (Figure 4), was the primary flavonoid identified in flower *S. canadensis* extracts, with concentrations of 30.70 mg/g DW in 70% ethanol and 19.65 mg/g DW in 80% methanol (Table 1). These findings align with those of Zekič et al. [35], who reported a rutin content of 27.62 ± 0.45 mg/g DW in 70% methanol extracts of inflorescences. Furthermore, Shelepova et al. [17] identified rutin as a major compound in aerial parts of *S. canadensis* extracted with methanol, ethanol, and acetone (200.45–211.20 mg/g). The solubility of flavonoids and organic acids in alcoholic extracts is primarily influenced by solvent polarity. Apati et al. [50] demonstrated that 70% ethanol yielded the highest concentration of this compound at 572.5 mg/L. Quercetin-3-rutinoside showed significant antibacterial activity and can be used as a natural antibiotic to treat different infectious diseases [51]. Quercetin glycosides, such as rutin, can interact with ammonium while entering the rhizosphere through plant exudates. As a result, new compounds, such as the phenol-ammonia complex, are formed. At low concentrations (20 μg/mL), these substances promote the development of lateral and adventitious roots in plants, and in concentrations higher than 100 μg/mL, inhibit them [52]. The goldenrods are also known for the rapid uptake of NO_3_–N and phosphorus, which can halt the development of co-occurring plants [53].

Furthermore, the flower extract was rich in flavonoid isorhamnetin-3-rutinoside, known as narcissin (Figure 4). It is naturally synthesized in plants via the phenylpropanoid pathway, and its production is triggered by environmental stressors such as UV radiation [54]. Studies have shown that isorhamnetin exhibits a wide range of pharmacological effects on cardiovascular diseases [55] and various types of tumors [56]. Additionally, it holds the potential to prevent neurodegenerative diseases such as Alzheimer’s disease [57]. This compound has garnered significant attention recently due to its widespread availability, affordability, high effectiveness, low toxicity, and minimal side effects [58].

Kaempferol-3-rutinoside, also known as nicotiflorin (Figure 4), was identified in both leaf and flower extracts. The measured concentrations were 7.92 mg/g DW in ethanolic and 6.79 mg/g DW in methanolic leaf extracts, while in flower extracts, the values were 5.41 mg/g DW for ethanolic and 4.19 mg/g DW for methanolic extracts. Kaempferol is a low molecular weight flavonoid that plants use to stimulate and regulate their growth as well as for defense purposes [59]. The bioassay results demonstrated that flavonoid nicotiflorin has a neuroprotective effect [60], protective effects on reducing memory dysfunction [61], and antioxidant activities [62]. It is often referred to as a compound with multifaceted therapeutic potential, known for its anti-inflammatory properties, hepatoprotective effects, anti-cancer activity, wound healing capabilities, and cardioprotective benefits [63].

Furthermore, leaf (0.816 ± 0.106 and 0.454 ± 0.072 mg/g DW) and flower (0.734 ± 0.160 and 0.699 ± 0.056 mg/g DW) samples in both solvents contained quercetin-3-galactoside, known as hyperoside. This compound is recognized by the European Pharmacopoeia as a standard used for calculating flavonoid content in *Solidaginis* herba. Similarly, in the research of Avertseva et al. [34], this compound was predominant in leaves in 50% ethanol (8.39 ± 0.60 mg/g). The difference in content can be explained by the difficulty of extracting this compound due to its chemical complexity, interactions with other plant matrix components, solubility challenges, and sensitivity to environmental conditions [64].

During the preparation of leaf and flower samples, foam formation was observed, suggesting the potential presence of saponin compounds. The extraction of saponins can be challenging due to their high polarity and molecular weight [65]. Given the established abundance of saponins in *S. canadensis* extracts [31,66] and their recognized role in plant defense against herbivory and pathogens [67], further investigation into the isolation and characterization of these compounds from this species is needed.

Given the pharmaceutical industry’s ongoing search for novel phytochemicals, *S. canadensis* emerges as a promising candidate. The plant’s substantial antioxidant capacity provides a strong foundation for developing high-value phytopharmaceutical products. Moreover, its widespread distribution across Europe presents a considerable resource for exploitation [68]. Phenolic compounds and essential oils are recognized for their antioxidant, antimicrobial, and antifungal properties, making them key constituents in developing phytotherapeutic drugs for chronic disease management [69,70]. The leaf extracts of *S. canadensis* were declared to have promising potential in the green synthesis of gold nanoparticles used in medicine [71]. Mariychuk et al. [71] showed that the extract of *S. canadensis* with its specialized metabolites can act both as a reducing agent and as a stabilizing agent for noble metal nanoparticles.

Most studies on *S. canadensis* focus on the phytochemical content or biological activity of its underground and aerial parts. Our study provides a novel comparison of leaves and flowers (separated from the inflorescence) in terms of phenolic content and antioxidant activity, representing the first targeted analysis of this kind. Our study reveals significant differences in phytochemical profiles between flowers and leaves, providing new insights into the species’ biological activity.

We present the site-specific phenolic profile of this invasive species in the context of the particular potential use of this species. By assessing the antioxidant capacity of extracts from different plant parts and evaluating various extraction solvents, our findings establish a basis for future research into its potential applications locally and beyond.

## 3. Materials and Methods

### 3.1. Plant Material

The leaves and inflorescences of *S. canadensis* were collected during the vegetation year 2021, from June to September, in the Istria region (Croatia). The 15 samples were gathered from three distinct locations (5 per each location), spanning latitudes from N 45.4073056 to N 44.8461944. The plant material underwent air-drying in the dark at room temperature after harvest. Before the grinding and extraction process, we separated the individual flowers from the larger clusters, known as inflorescences. To ensure clarity, we used the term “flower” extracts throughout this paper.

### 3.2. Extraction Procedure

The dry plant material of each sample (250 g) was pooled to create a representative sample for the study area. The material was minced using the Grindomix GM 200 knife mill, programmed at 10,000×/30 s (Retsch, Haan, Germany).

To spectrophotometrically determine phenolic content, extracts were prepared in quadruplicate in three repetitions following a standardized protocol of Bilić et al. [72]. Within the tubes, 0.06 g of plant material was dissolved in 2 mL of solvent (70% EtOH and 80% MeOH). These prepared solutions were sonicated for 30 min in an ultrasonic bath (40 kHz, 300 W ultrasound power, 400 W heater power, Holon, Israel), followed by centrifugation at 12,000 rpm/10 min (Jouan MR23i, Jouan S.A., Saint-Herblain, France) and filtration through 0.20 µm polytetrafluoroethylene filters (Macherey-Nagel, Düren, Germany) before being stored at +4 °C until analysis.

### 3.3. HPLC-DAD-MS Analysis of Phenolic Compounds in Leaf and Flower Extracts

The extraction of phenolic compounds for identification via HPLC-DAD-MS was performed following the protocol of Mikulič-Petkovšek et al. [73]. The 0.2 g of dried plant tissue was extracted with 6 mL of 70% EtOH and 80% MeOH containing 3% (*v*/*v*) formic acid in a cooled ultrasonic bath for 60 min. Extracts were centrifuged for 10 min at 10,000× *g* and filtered through 20 µm polytetrafluoroethylene (PTFE) filters (Macherey-Nagel, Düren, Germany). All extracts were subjected to LC-DAD-MS analysis to identify and quantify specific phenolic compounds. Analyte separation was performed using HPLC (Dionex UltiMate 3000, Thermo Fisher Scientific, San Jose, CA, USA) with a DAD detector, maintaining a column temperature (Gemini C18, Phenomenex, Torrance, CA, USA) at 25 °C. Compounds were detected at wavelengths of 280 and 350 nm. Two mobile phases were used for the separation of phenolic compounds: mobile phase A consisted of double-distilled water/acetonitrile/formic acid (96.9/3/0.1, *v*/*v*/*v*), and mobile phase B was double-distilled water/acetonitrile/formic acid (3/96.9/0.1, *v*/*v*/*v*). The elution followed a linear gradient: from 5% to 20% B over the first 15 min, from 20% to 30% B in the next 5 min, then an isocratic mixture for 5 min, followed by a gradient from 30% to 90% B in 5 min, and an isocratic mixture for 15 min before reverting to initial conditions, as per the method described by Mikulic-Petkovsek et al. [74].

The sample injection volume was 20 μL, with a mobile phase flow rate of 0.6 mL/min. Individual metabolites were identified using mass spectrometry (LTQ XL Linear Ion Trap Mass Spectrometer, Thermo Fisher Scientific, San Jose, CA, USA) with electrospray ionization (ESI) in a negative scanning mode, following modified parameters from the study of Mikulic-Petkovsek et al. [73]. The scanning range was from *m*/*z* 110 to 1700—a data-dependent full scan. Phenolic compounds were confirmed based on fragmentation products, retention times of corresponding standards, and the spectral comparison of individual peaks with those of the standards. The share of each phenolic compound was determined by analyzing the peak areas of the samples against the corresponding standard curves and was expressed in mg/g DW. The following external standards were used: caffeic acid, apigenin-7-glucoside, ferulic acid, quercetin-3-O-rhamnoside, neochlorogenic (3-caffeoylquinic) acid, naringenin, ellagic acid, gallic acid, chlorogenic acid, and rutin (quercetin-3-O-rutinoside); (-)epicatechin, quercetin-3-O-galactoside, quercetin-3-O-glucoside, *p*-coumaric acid, procyanidin B1, and kaempferol-O-glucoside; quercetin-3-O-xyloside and quercetin-3-O-arabinopyranoside; and isorhamnetin-3-O-glucoside.

### 3.4. Total Phenolic, Flavonoid, and Non-Flavonoid Content and Antioxidant Capacity

Total phenolics (TP) were determined using the method of Singleton and Rossi [75], while total non-flavonoids (TNF) were measured using the method described by Ough and Amerine [76]. Both methods rely on the reduction in the Folin–Ciocalteu (FC) reagent in the presence of phenolics, resulting in the formation of molybdenum-tungsten blue, which is then measured spectrophotometrically at 765 nm. In the TP assay, 0.1 mL of extract was mixed with 1.5 mL of distilled water and 0.1 mL of the Folin–Ciocalteu (FC) reagent. The reaction was allowed to proceed for 5 min at room temperature. Following this, 1.5 mL of 20% sodium carbonate solution was added to the mixture, and the reaction was allowed to develop for 30 min at room temperature. The formation of the molybdenum-tungsten blue complex was measured spectrophotometrically at 765 nm. In the TNF assay, a 0.1 mL sample of the extract was mixed with 1.5 mL of distilled water and 0.1 mL of the FC reagent. The reaction was allowed to develop for 5 min at room temperature. Following this, 1.5 mL of 20% sodium carbonate solution was added and incubated for 30 min at room temperature. The resulting blue complex was quantified spectrophotometrically at 765 nm. The results were calculated according to the calibration curve for gallic acid (y = 0.004x, R^2^ = 0.991 for 70% ethanol extracts and y = 0.009x, R^2^ = 0.996 for 80% methanol extracts, where y is the absorbance at 765 nm and x is the concentration of gallic acid mg/L) and expressed as mg of gallic acid equivalents (GAE) per g of dry weight (DW).

Total flavonoid (TF) content was measured following the protocol described by Martins et al. [77]. In the TF assay, to a 1 mL sample of the extract, 1 mL of 2% aluminum chloride (AlCl_3_) in ethanol was added. After 15 min of incubation at room temperature, the absorbance of the reaction mixture was measured spectrophotometrically at 430 nm. The results were calculated according to the calibration curve for catechin (y = 0.0024x, R^2^ = 0.993 for 70% ethanol extracts and y = 0.0022x, R^2^ = 0.994 for 80% methanol extracts, where y is the absorbance at 765 nm and x is the concentration of gallic acid mg/L) and expressed as mg of (+)-catechin equivalents (CE) per g of dry weight (DW).

The antioxidant capacity (AC) of the extracts was determined spectrophotometrically using standard DPPH, ABTS, and FRAP assays following the methods described in the study of Poljuha et al. [78]. The DPPH assay was performed by mixing 0.1 mL of the extract with 3.9 mL of DPPH radical solution (0.1 mM in methanol). After 30 min of incubation in the dark at room temperature, the absorbance of the mixture was measured at 517 nm. The results of DPPH analysis were calculated against a Trolox calibration curve (y = 44.991x, R^2^ = 0.972 for 70% ethanol extracts; y = 47.786x, R^2^ = 0.992 for 80% methanol extracts) and expressed as mg of Trolox equivalents (TE) per g of dry weight (mg TE/g DW). The FRAP assay was carried out by mixing 0.1 mL of extract with 3 mL of FRAP reagent (containing 10 mM 2,4,6-tripyridyl-s-triazine in 40 mM HCl, 20 mM FeCl_3_, and 300 mM acetate buffer, pH 3.6). The mixture was incubated for 30 min at room temperature, and the absorbance was measured at 593 nm. FRAP values were calculated using a Trolox calibration curve (y = 1.638x, R^2^ = 0.993 for 70% ethanol extracts; y = 1.586, R^2^ = 0.991 for 80% methanol extracts) and as mg of Trolox equivalents (TE) per g of dry weight (mg TE/g DW). For the ABTS assay, the ABTS radical cation was generated by reacting ABTS solution (7 mM) with potassium persulfate (2.45 mM) and incubating the mixture in the dark for 12 h at room temperature. The radical cation was then diluted with ethanol to obtain an absorbance of 0.70 ± 0.02 at 734 nm. A 100 µL aliquot of the extract was mixed with 3.9 mL of the ABTS solution, and after 6 min, the absorbance was measured at 734 nm. ABTS values were calculated using a Trolox calibration curve (y = 46.137x, R^2^ = 0.981 for 70% ethanol extracts; y = 41.432, R^2^ = 0.968 for 80% methanol extracts) and as mg of Trolox equivalents (TE) per g of dry weight (mg TE/g DW).

All measurements were performed in triplicate using a NanoPhotometer P300 spectrophotometer (Implen GmbH, München, Germany) adjusted to a 2 mL cuvette volume.

### 3.5. Statistical Analysis

Two-way analysis of variance (ANOVA) with post hoc Tukey’s test was conducted to determine the significance of the factors and to assess the significance of differences between the extracts (*p* ≤ 0.05 and 0.01). Pearson’s correlation coefficients were calculated to assess the interaction between bio-compounds and antioxidant capacity. Data were statistically analyzed using the software Statgraphics Plus 4.0 (Manugistics, Inc., Rockville, MD, USA) and IBM SPSS 23 (Chicago, IL, USA). Visualization (Figure 1, Figure 2 and Figure 3) was generated using Flourish Studio 1.0.0.3 (Canva, Sidney, Australia) and Sketch version 101 (Eindhoven, The Netherlands).

## 4. Conclusions

This study presents a comprehensive site-specific analysis of the phenolic profile and antioxidant capacity of *Solidago canadensis* leaf and flower extracts. The results reveal that leaf extracts exhibit higher diversity in hydroxycinnamic acids (21 compounds) and flavonols (17 compounds), while flower extracts demonstrate a similar distribution, with 19 hydroxycinnamic acids and 15 flavonols identified. Key compounds include 5-caffeoylquinic acid, quercetin-3-rutinoside, kaempferol-3-rutinoside, and isorhamnetin-3-rutinoside, with notable organ-specific variations in concentration. Ethanolic extracts were more efficient than methanolic extracts in extracting total phenolics (TP) and non-flavonoids (TNF), demonstrating higher yields across both plant parts. Antioxidant capacity, assessed through ABTS, DPPH, and FRAP assays, was higher in flowers than leaves, suggesting significant plant part-specific antioxidant potential. Strong positive correlations were observed between TP, TNF, and antioxidant capacities, further validating the contribution of these compounds to the biological activity of *S. canadensis*.

The findings underscore the potential of S. *canadensis* as a valuable source of bioactive phenolics, particularly for phytopharmaceutical applications. Moreover, the differentiation in phenolic composition between plant parts offers insights into optimizing extraction for specific bioactive compounds. These results open opportunities for further research into using invasive species as a source of antioxidants and therapeutic agents, offering a balance between ecological management and potential pharmaceutical applications.

## Figures and Tables

**Table 1:** Phenolic compounds (mg/g of dry weight (DW)) of *S. canadensis* leaf and flower extracts in 70% ethanol and 80% methanol extracts identified by LC-MS. Values represent the mean ± SD of three replicates. The total phenolic contents of the main phenolic groups are given in bold. Different letters (a–d) in the same row indicate significant differences (ANOVA, Tukey’s honest significant difference, *p*-value ≤ 0.01; * *p*-value ≤ 0.01); n.d.—not detected.

Phenolic Compounds	*Solidago canadensis* Leaf		*Solidago canadensis* Flower	
	70% EtOH	80% MeOH	70% EtOH	80% MeOH
3-caffeoylquinic acid 1	1.393 ± 0.092 ^a^	1.270 ± 0.124 ^a^	0.433 ± 0.161 ^b^	0.617 ± 0.071 ^b^
4-caffeoylquinic acid 1	0.542 ± 0.098 ^a^	0.653 ± 0.123 ^a^	0.383 ± 0.096 ^a^	0.477 ± 0.121 ^a^
5-caffeoylquinic acid 1	17.748 ±1.558 ^a^	20.423 ± 0.737 ^a^	9.837 ± 0.685 ^c^	13.600 ± 0.667 ^b^
5-caffeoylquinic acid 2	0.920 ± 0.053 ^a^	0.797 ± 0.078 ^a^	0.732 ±0.200 ^ab^	0.413 ± 0.088 ^b^
Caffeic acid	0.008 ± 0.000 ^c^	0.007 ± 0.000 ^c^	0.172 ± 0.047 ^a^	0.097 ± 0.021 ^b^
Caffeic acid hexoside 1	0.652 ± 0.013 ^a^	0.293 ± 0.017 ^b^	0.766 ± 0.128 ^a^	0.532 ± 0.088 ^ab^
Caffeic acid hexoside 2	0.006 ± 0.001 ^a^	0.006 ± 0.001 ^a^	0.008 ± 0.001 ^a^	0.009 ± 0.002 ^a^
Dicaffeoylquinic acid 1	0.349 ± 0.023 ^c^	0.225 ± 0.014 ^c^	0.930 ± 0.337 ^ab^	1.538 ± 0.244 ^a^
Dicaffeoylquinic acid 2	2.412 ± 0.058 ^b^	2.868 ± 0.018 ^a^	n.d.	n.d.
Dicaffeoylquinic acid 3	0.149 ± 0.014 ^a^	0.130 ± 0.013 ^a^	0.237 ± 0.117 ^a^	0.275 ± 0.015 ^a^
Dicaffeoylquinic acid 4	0.344 ± 0.042 ^c^	0.463 ± 0.024 ^c^	0.965 ± 0.124 ^b^	1.247 ± 0.088 ^a^
*p*-coumaric acid	0.486 ± 0.007 ^a^	0.169 ± 0.006 ^b^	0.124 ± 0.040 ^bc^	0.069 ± 0.011 ^c^
*p*-coumaric acid hexoside 1	0.261± 0.026 ^a^	0.114 ± 0.013 ^b^	0.069 ± 0.011 ^c^	0.048 ± 0.008 ^c^
*p*-coumaric acid hexoside 2	0.173 ± 0.010 ^a^	0.188 ± 0.012 ^a^	0.001 ± 0.000 ^b^	0.001 ± 0.000 ^b^
3-*p*-coumaroylquinic acid	1.694 ± 0.554 ^a^	0.468 ± 0.080 ^b^	0.752 ± 0.071 ^b^	0.119 ± 0.024 ^b^
4-*p*-coumaroylquinic acid 1	0.293 ± 0.018 ^a^	0.088 ± 0014 ^b^	n.d.	n.d.
5-*p*-coumaroylquinic acid 1	0.556 ± 0.036 ^a^	0.572 ± 0.046 ^a^	0.264 ± 0.062 ^b^	0.200 ± 0.051 ^b^
5-*p*-coumaroylquinic acid 2	0.290 ± 0.033 ^a^	0.381 ± 0.012 ^a^	0.273 ±0.138 ^a^	0.234 ± 0.039 ^a^
3-feruloylquinic acid	0.081 ± 0.014 ^ab^	0.092 ± 0.019 ^a^	0.034 ± 0.009 ^c^	0.043 ± 0.011 ^bc^
5-feruloylquinic acid 1	1.013 ± 0.095 ^ab^	1.225 ± 0.060 ^a^	0.805 ± 0.056 ^bc^	0.750 ± 0.130 ^c^
Ferulic acid	0.002 ± 0.000 ^b^	0.002 ± 0.001 ^b^	0.191 ± 0.045 ^a^	0.145 ± 0.037 ^a^
**Hydroxycinnamic acid** **derivatives**	**27.749 ± 1.081 ^a^**	**28.594 ± 3.694 ^a^**	**17.272 ± 1.480 ^b^**	**20.498 ± 0.944 ^b^**
*p*-hydroxybenzoic acid	0.591 ± 0.023 ^a^	0.213 ± 0.027 ^b^	n.d.	n.d.
Syringic acid	0.713 ± 0.021 ^b^	0.417 ± 0.009 ^c^	0.901 ± 0.002 ^a^	0.224 ± 0.033 ^d^
Protocatechuic acid	0.023 ± 0.002 ^b^	0.021 ± 0.002 ^b^	0.526 ± 0.024 ^a^	0.535 ± 0.008 ^a^
**Hydroxybenzoic acid** **derivatives**	**1.328 ± 0.020 ^a^**	**0.645 ± 0.020 ^c^**	**1.427 ± 0.023 ^b^**	**0.759 ± 0.023 ^d^**
Quercetin pentoside 1	0.045 ± 0.005 ^a^	0.036 ± 0.003 ^a^	0.041 ± 0.014 ^a^	0.037 ± 0.001 ^a^
Quercetin pentoside 2	0.324 ± 0.021 ^a^	0.210 ± 0.013 ^b^	0.056 ± 0.020 ^c^	0.092 ± 0.015 ^c^
Quercetin-3-rutinoside	14.465 ± 0.265 ^bc^	11.317 ± 0.559 ^c^	30.702 ± 4.158 ^a^	19.653 ± 2.434 ^b^
Quercetin-3-galactoside	0.816 ± 0.106 ^a^	0.454 ± 0.072 ^b^	0.734 ± 0.160 ^ab^	0.699 ± 0.056 ^ab^
Quercetin-3-glucoside	0.710 ± 0.018 ^c^	0.684 ± 0.058 ^c^	4.881 ± 0.442 ^a^	4.020 ± 0.223 ^b^
Quercetin-3-rhamnoside	0.127 ± 0.005 b	0.157 ± 0.016 a	n.d.	n.d.
Quercetin acetylhexoside 1	3.210 ± 0.258 ^b^	3.957 ± 0.017 ^a^	n.d.	n.d.
Quercetin pentosylhexoside	0.578 ± 0.090 ^b^	0.252 ± 0.069 ^b^	1.064 ± 0.171 ^a^	1.242 ± 0.162 ^a^
Isorhamnetin hexoside	0.827 ± 0.000 ^a^	1.226 ± 0.677 ^a^	0.382 ± 0.144 ^b^	0.159 ± 0.018 ^b^
Isorhamnetin pentosylhexoside	0.395 ± 0.044 ^b^	0.438 ± 0.046 ^b^	0.537 ± 0.137 ^b^	1.210 ± 0.062 ^a^
Isorhamnetin-3-rutinoside	3.765 ± 0.327 ^b^	3.781 ± 0.502 ^b^	6.034 ± 0.452 ^a^	6.548 ± 0.393 ^a^
Isorhamnetin acetylhexoside	1.489 ± 0.151 ^b^	1.590 ± 0.128 ^b^	2.436 ± 0.332 ^a^	2.123 ± 0256 ^a^
Kaempferol rhamnosylhexoside 1	2.275 ± 0.058 ^a^	2.100 ± 0.058 ^a^	1.514 ± 0.137 ^b^	1.248 ± 0.069 ^c^
Kaempferol-3-galactoside	0.749 ± 0.089 ^a^	0.601 ± 0.051 ^ab^	0.450 ± 0.157 ^b^	0.408 ± 0.016 ^b^
Kaempferol-3-rutinoside	7.921 ± 0.340 ^a^	6.792 ± 0.465 ^a^	5.411 ± 0.555 ^b^	4.189 ± 0.254 ^c^
Kaempferol-3-glucoside	2.120 ± 0.203 ^a^	1.430 ± 0.207 ^b^	0.937 ± 0.354 ^bc^	0.391 ± 0.044 ^c^
Kaempferol acetylhexoside 1	4.595 ± 0.306 ^a^	4.277 ± 0.450 ^a^	1.851 ± 0.102 ^b^	1.982 ± 0.154 ^b^
**Flavonols**	**43.580 ± 1.392 ^b^**	**38.686 ± 1.699 ^b^**	**57.028 ± 4.649 ^a^**	**44.000 ± 3.774 ^b^**
**TOTAL**	73.063 ± 1.553 ^ab^*	67.924 ± 3.701 ^ab^*	75.723 ± 5.836 ^a^*	65.257 ± 4.702 ^b^*

## Data Availability

Data are presented in the manuscript.

## Supplementary Materials

The following supporting information can be downloaded at https://www.mdpi.com/article/10.3390/plants14010044/s1, Table S1: The concentrations of total phenolics (TP), total non-flavonoids (TNF), total flavonoids (TF), and antioxidant capacity (obtained by DPPH, ABTS, and FRAP assays) in *Solidago canadensis* L. leaf and flower extracts in two solvents; Table S2: Pearson’s correlation coefficients (two-tailed) between total phenolic (TP), total non-flavonoids (TNF), and total flavonoids (TF) contents and antioxidant capacity (obtained by DPPH, ABTS, and FRAP assays).
