# Supplementary material for: Phenolic Profile and Antioxidant Capacity of Invasive Solidago canadensis L.: Potential Applications in Phytopharmacy
Source: Plants (Basel). 2024 Dec 26;14(1):44. doi: 10.3390/plants14010044 (PMC11723282; doi:10.3390/plants14010044)
Supplement: Supplementary file 1 [file plants-14-00044-s001.zip › Table S1.pdf]

Table S1. The concentrations of total phenolics (TP), total non-flavonoids (TNF), total flavonoids (TF), and antioxidant capacity (obtained by DPPH, ABTS, and FRAP assays) in *Solidago canadensis* L. leaf and flower extracts in two solvents: SL, *S. canadensis* leaf extract; SF, *S. canadensis* flower extract; EtOH, ethanol; MeOH, methanol.

	**Plant organ**	**Solvent**	
		**70% EtOH**	**80% MeOH**
TP (mg GAE/g DW)	SL	110.68 ± 4.87 [a]	49.45 ± 1.17 [b]
	SF	110. 77 ± 5.94 [a]	45.78 ± 2.62 [b]
TNF (mg GAE/g DW)	SL	72.20 ± 2.82 [a]	29.68 ± 0.64 [b]
	SF	64.70 ± 5.18 [a]	28.38 ± 1.38 [b]
TF (mg CE/g DW)	SL	29.27 ± 3.94 [a]	33.87 ± 1.03 [a]
	SF	34.14 ± 3.04 [a]	30.38 ± 2.72 [a]
DPPH (mg TE/g DW)	SL	73.81 ± 4.32 [ab]	60.98 ± 3.46 [b]
	SF	93.06 ± 8.67 [a]	89.75 ± 10.08 [a]
ABTS (mg TE/g DW)	SL	54.61 ± 10.23 [b]	53.76 ± 4.61[b]
	SF	81.86 ± 4.10 [a]	83.82 ± 7.27 [a]
FRAP (mg TE/g DW)	SL	63.24 ± 0.71 [ab]	60.62 ± 1.02 [b]
	SF	68.82 ± 2.06 [c]	66.98± 5.17 [ab]

Data are presented as mean ± SD; mean values within one method followed by different letters (a–d) are significantly different at the 1% level of probability (ANOVA, Tukey test, $p \leq 0.01$).
