# Supplementary material for: Phenolic Profile and Antioxidant Capacity of Invasive Solidago canadensis L.: Potential Applications in Phytopharmacy
Source: Plants (Basel). 2024 Dec 26;14(1):44. doi: 10.3390/plants14010044 (PMC11723282; doi:10.3390/plants14010044)
Supplement: Supplementary file 1 [file plants-14-00044-s001.zip › Table S2.pdf]

Table S2. Pearson's correlation coefficients (two-tailed) between total phenolic (TP), total non-flavonoids (TNF), and total flavonoids (TF) contents and antioxidant capacity (obtained by DPPH, ABTS, and FRAP assay).

Correlation	TNF (mg GAE/g DW)	TF (mg CE/g DW)	FRAP (mg TE/g DW)	ABTS (mg TE/g DW)	DPPH (mg TE/g DW)
TP (mg GAE/g DW)	0,978 **	-0,089	0,252	-0,032	0,256
TNF (mg GAE/g DW)		0,026	-0,215	-0,111	-0,195
TF (mg CE/g DW)			0,063	-0,082	-0,063
FRAP (mg TE/g DW)				0,825 **	0,850 **
ABTS (mg TE/g DW)					0,849 **

** significant correlation at the 1% level of probability ($p \leq 0.01$).
